# Quantitative and correlation analysis of the DNA methylation and expression of DAPK in breast cancer

**DOI:** 10.7717/peerj.3084

**Published:** 2017-03-14

**Authors:** Youzhi Zhu, Shuiqin Li, Qingshui Wang, Ling Chen, Kunlin Wu, Yide Huang, Xiangjin Chen, Yao Lin

**Affiliations:** 1 Department of Thyroid and Breast Surgery, The First Affiliated Hospital of Fujian Medical University, Fuzhou, China; 2 College of Life Sciences, Fujian Normal University, Fuzhou, China

**Keywords:** DAPK, Breast cancer, DNA methylation, Correlation

## Abstract

**Background:**

Death-associated protein kinase 1 (DAPK) is an important tumor suppressor kinase involved in the regulation of multiple cellular activities such as apoptosis and autophagy. DNA methylation of DAPK gene was found in various types of cancers and often correlated with the clinicopathological characteristics. However, the mRNA and protein expression of DAPK in the same sample was rarely measured. Thus, it was unclear if the correlation between DAPK gene methylation and clinicopathological parameters was due to the loss of DAPK expression.

**Methods:**

In this study, the DNA methylation rate, mRNA and protein expression of DAPK was quantitatively detected in 15 pairs of breast cancer patient samples including tumor (T) and adjacent non-tumor (N) tissues.

**Results:**

The correlation between DNA methylation rate and mRNA expression, together with the correlation between mRNA and protein expression, was calculated. No correlation was observed between any levels using either the measurement value of each sample or the T/N ratio of each pair.

**Discussion:**

These data suggested that the DNA methylation status of DAPK did not correlate well with its mRNA or protein expression. Extra caution is needed when interpreting the DNA methylation data of DAPK gene in clinical studies.

## Introduction

Death-associated protein kinase 1 (DAPK) is an important tumor suppressor kinase that participates in apoptosis, autophagy, cell migration and so on (Lin, Hupp & Stevens, 2010; Schneider-Stock, 2014; Shiloh, Bialik & Kimchi, 2014). The expression of DAPK is often lost in various types of tumor due to DNA hypermethylation (Mittag et al., 2006; Shohat et al., 2002). The DNA methylation of DAPK was found to be correlated with multiple clinicopathological characteristics in many cancer types (Kaufmann & Earnshaw, 2000; Tang et al., 2000). However, in the over 100 studies on DAPK methylation in cancer in the past two decades, only few of them detected the mRNA or protein levels of DAPK in the same patient samples (Huang et al., 2014). It was unclear whether the hypermethylation of DAPK gene actually correlate with down-regulation of DAPK mRNA or protein expression. Therefore, it is important to investigate the DNA methylation and expression of DAPK in the same patient samples for a more accurate mechanistic explanation of the clinical significance of DAPK DNA methylation.

DNA methylation of DAPK in breast cancer was examined in several studies (Lehmann et al., 2002; Van der Auwera et al., 2009, 2010). However, the protein expression of the matched samples was not tested in these studies. Hence, it was not yet clear if the DNA methylation status of DAPK in breast cancer correlated with its mRNA or protein expression. Since 1990s, the incidence of breast cancer in China has increased dramatically at a speed twice as fast as the rest of the world. At present, breast cancer is the most common female cancer and ranked sixth for cancer-associated death in China (Fan et al., 2014). It will be interesting to compare the methylation status and expression levels of DAPK in a Chinese-patient cohort.

In this study, we employed a quantitative method to measure the DNA methylation of DAPK gene together with the expression DAPK mRNA and protein in 15 pairs of breast cancer patient samples. The correlation between the DAPK DNA methylation rate, mRNA expression and protein levels in these samples were investigated.

## Materials and Methods

### Clinical samples

The 15 pairs of breast cancer patient samples were obtained from patients with invasive ductal carcinoma at The First Affiliated Hospital of Fujian Medical University. Female patients diagnosed with primary breast cancer at AJCC stage I to III and underwent surgical resection of tumor were selected. When the breast specimen was ex vivo, first we obtained the normal breast tissue (0.5 × 1 cm) around 3–5 cm outside the tumor area. If it was a breast-conserving surgery, we obtained specimens from the edge of the preserved breast. Then, we obtained simple cancer tissue (0.5 × 0.5 cm or 0.5 × 1 cm) according to the tumor size. Specimens were rapidly placed into freezing tubes without RNAse and preserved in liquid nitrogen. The whole procedure was performed within 3 min to avoid cross-contamination. Written consent was obtained for all patients. For the biological replicate (i.e., patient samples), we only had one piece of samples from each patient for DNA, mRNA and protein detection mentioned below. For each detection method, the technical replicates were performed in triplicates. Medical ethics committee of The First Affiliated Hospital of Fujian Medical University approval to carry out the study within its facilities (approval number: [2015]108).

### DNA extraction and bisulfite modification

Genomic DNA from frozen specimens with the weight about 30–60 mg was extracted by phenol/chloroform/isoamyl alcohol extraction and ethanol precipitation, dissolved in 50–100 ml of distilled water and stored at −20 °C until usage. About 1 μg of genomic DNA was subject to sodium bisulfite modification to convert unmethylated cytosines to uracils. All the procedures were performed according to the CpGenome™ Universal DNA Modification Kit (Millipore, Billerica, MA, USA) before the samples were subject to polymerase chain reaction (PCR).

### Construction of plasmid “pUC57-methyl”

**Figure 1:**
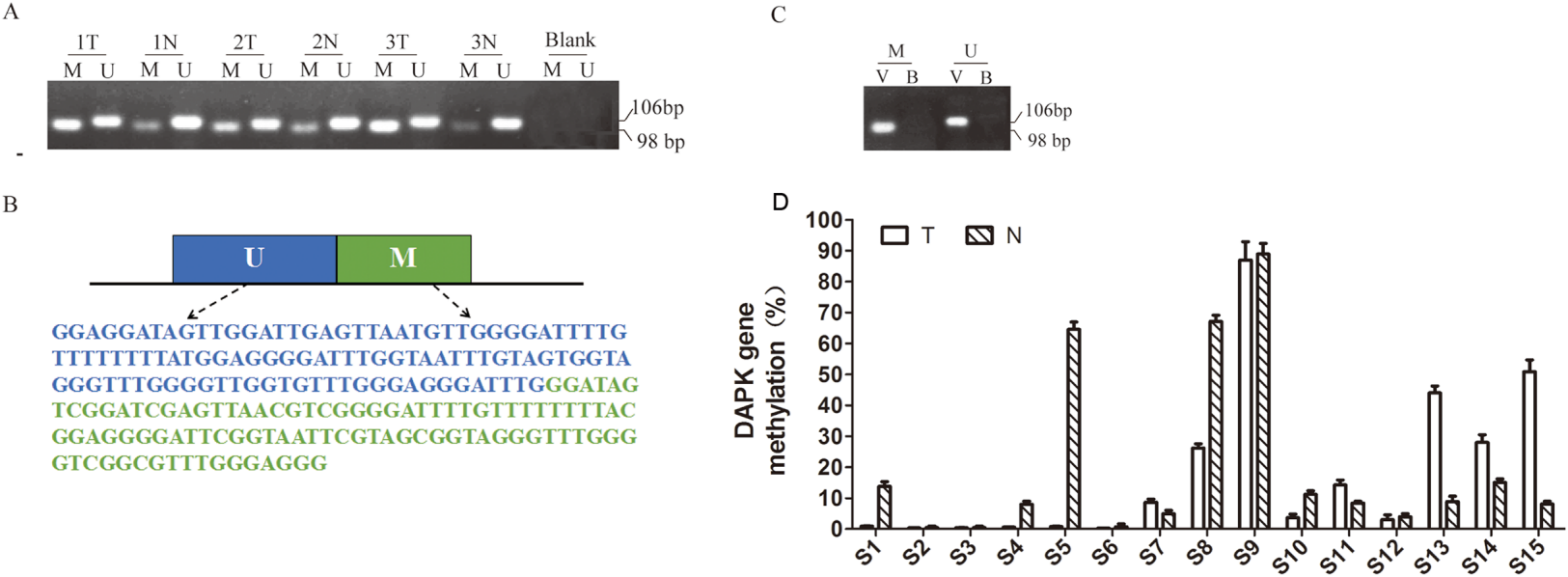
Quantitative detection of *DAPK* promoter methylation in breast cancer samples. (A) Detection of DAPK promoter methylation in three pairs of random breast cancer patient samples. The methylation-specific PCR products were resolved in 2% agarose gels. M, methylated *DAPK*; U, unmethylated *DAPK*; Blank, negative control (without DNA); T, the breast cancer tissue; N, the corresponding adjacent normal tissue. (B) The PCR product sequences inserted into synthetic plasmid. UM, sequence for unmethylated *DAPK* gene promoter; M, sequence for methylated *DAPK* genepromoter. (C) Validation of the engineered construct as PCR template. B, blank; V, vector. (D) Quantitative detection of *DAPK* promoter methylation in breast cancer samples using real-time PCR and. T, breast cancer tumor tissue; N, the corresponding adjacent normal tissue; S1–S15, patient codes.

The sequences of the PCR products for both the M and U primers (Fig. 1B) are cloned into the plasmid pUC57 and named pUC57-methyl (Sangon Biotech, Shanghai, China).

### Methylation-specific PCR

In a typical experiment, 5 μl modified DNA or 1 μl of synthesized plasmid was used as the template in a total volume of 25 μl. The methylation-specific PCR (MSP) solution contained 1 × EX *Taq* buffer, 2.5 mM dNTP 2 μl, 10 μM PCR primers 0.5 μl and 0.125 μl EX *Taq* HS DNA polymerase (TaKaRa, Tokyo, Japan). PCR conditions are: 95 °C for 5 min, 35 cycles at 95 °C for 15 s, 55 °C for 15 s, 72 °C for 15 s and a 5 min extension was allowed at 72 °C.

### RNA extraction and detection

The mRNA from patient tissue was extracted using the Eastep™ Total RNA Extraction Kit (Promega, Beijing, China) and reverse transcribed using the GoScript™ Reverse Transcription System (Promega, Madison, WI, USA) according to the manufacturers’ instruction. The real-time PCR (RT PCR) primers for *DAPK* (NCBI reference sequence: NG_029883.1) and glyceraldehyde-3-phosphate dehydrogenase (*GAPDH*; NCBI reference sequence: NG_007073.2) were listed in Table 1. PCR reaction conditions were as follows: 95 °C for 2 min, 40 cycles at 95 °C for 15 s and 60 °C for 1 min. The cDNA from HEK293T cells were used as a positive control and formation of the standard curve.

**Table 1:** Summary of primer sequences and product size used in the PCR procedure.

Gene name	Sequences (5′–3′)	Product size (bp)
DAPK methylated	F: GGATAGTCGGATCGAGTTAACGTC	98
	R: CCC TCC CAA ACG CCG A	
DAPK unmethylated	F: GGAGGATAGTTGGATTGAGTTAATGTT	106
	R: CAAATCCCTCCCAAACACCAA	
DAPK RT PCR	F: GATAGAAATGTCCCCAAACCTCG	189
	R: TCTTCTTTGGATCCTTGACCAGAA	
GAPDH RT PCR	F: CGGAGTCAACGGATTTGGTCGTAT	304
	R: AGCCTTCTCCATGGTGGTGAAGAC	

**Note:**

RT PCR, real-time PCR; F, forward primer; R, reverse primer.

### Protein extraction and western blotting

The total protein from frozen specimens with the weight about 30–60 mg was extracted using 100–200 μl of radioimmunoprecipitation assay (RIPA) lysis buffer (Beyotime, Shanghai, China) containing protease inhibitors (Roche; Basel, Switzerland). Protein concentration was determined using BioTekDc Protein Assay (BioTek; Hercules, CA, USA). Equal amount of lysates (60–90 μg) were subject to SDS-PAGE. The primary anti-DAPK antibody is from LSBio (Life Span BioSciences Inc., Seattle, WA, USA). The horseradish peroxidase-conjugated goat anti-rabbit or anti-mouse secondary antibodies were from Sigma-Aldrich (St. Louis, MO, USA).

### Statistical analysis

Statistical analysis and visualization of the data was achieved using the GraphPad Prism software program (version, 6.04; GraphPad Software Inc., La Jolla, CA, USA). Data shown were presented as mean ± SD of three independent experiments, and the difference was considered statistically significant at *P* < 0.05 by using the Student’s *t*-test.

### Cell culture

Human embryonic kidney cells HEK293T were purchased from the Institute of Biochemistry and Cell Biology at the Chinese Academy of Sciences (Shanghai, China). The cell line has been verified by STR genotyping and tested negative for mycoplasma. HEK293T cell was maintained in DMEM (Invitrogen Corp., Carlsbad, CA, USA), containing 10% fetal bovine serum (FBS) (Equitech-Bio; Ingram, TX, USA), 100 units/ml penicillin G and 100 μg/ml streptomycin (Invitrogen Corp., Carlsbad, CA, USA). HEK293T cells were incubated at 37 °C in a humidified incubator with 5% CO_2_.

## Results

### Quantitative measurement of DAPK promoter hypermethylation

The first report on DAPK promoter hypermethylation was published in 1999 by Katzenellenbogen, Baylin & Herman (1999). Since then, most DNA methylation studies on DAPK gene was carried out using one set of MSP primers (Table 1) (Huang et al., 2014; Katzenellenbogen, Baylin & Herman, 1999). Therefore, we initially carried out the same MSP approach in three randomly picked pairs of breast cancer samples and successfully detected the methylated (M) and un-methylated (U) bands (Fig. 1A). However, it is difficult to quantitatively measure the methylation rate due to the differential cell composition in each sample and the binding affinity of the M and U primers. Therefore, we designed pUC57-methyl construct containing the PCR products for both the M and U primers (Fig. 1B) to allow quantitative measurement. The PCR reaction using pUC57-methyl as the template was clean, confirming the validity of this construct (Fig. 1C). Next, we measured the methylation rate of 15 pairs of breast cancer patient samples using RT PCR (Fig. 1D). The standard curve was created using the engineered construct. The methylation rate was calculated by comparing the PCR product of M against the total (M + U) for each sample, so that the problem of differential cell composition was avoided.

### Lack of correlation between DAPK mRNA expression and DNA methylation

**Figure 2:**
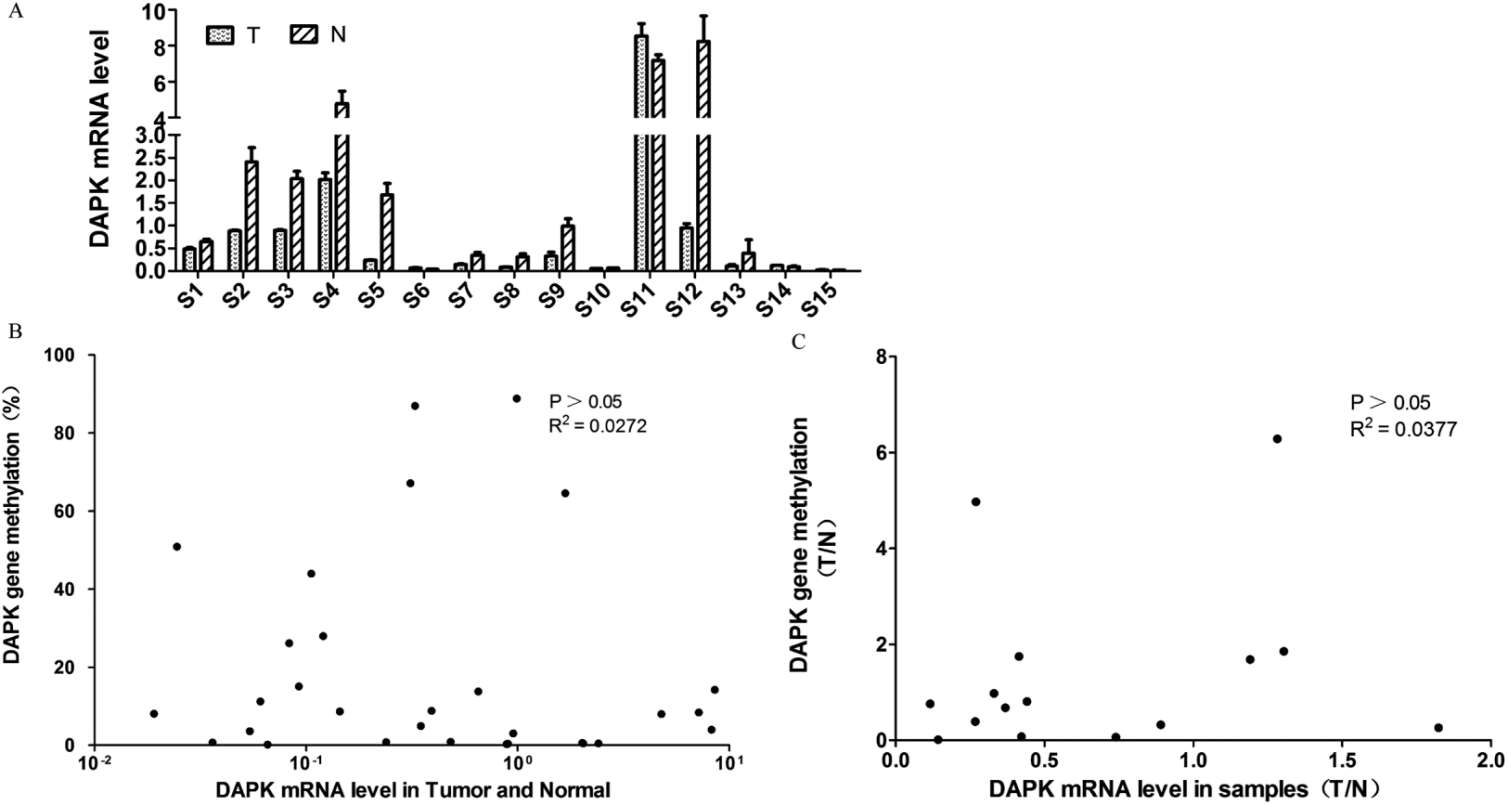
Analysis of the correlation between *DAPK* mRNA and promoter methylation in breast cancer samples. (A) The mRNA expression of *DAPK* was detected using real-time PCR. S1–S15, patient codes. The cDNA from HEK293T cells were used as a positive control and formation of the standard curve. (B) Correlation analysis of *DAPK* mRNA expression and *DAPK* promoter methylation rate in breast cancer samples. (C) Correlation analysis of the T/N ratio of *DAPK* mRNA expression and *DAPK* promoter methylation rate in breast cancer samples.

Next, the DAPK mRNA expression of these 15 pairs of breast cancer samples were analyzed using RT PCR (Fig. 2A). Similar to the DNA methylation data (Fig. 1D), differential expression was observed across the samples (Fig. 2A). In order to investigate whether the DNA methylation status correlate with the mRNA expression, a correlation analysis was carried out. No correlation was observed between DAPK DNA methylation rate and its mRNA expression in the total of 30 samples (Fig. 2B). Further, the tumor/normal (T/N) ratio of the 15 samples was used for the correlation analysis. Again no correlation was observed between the T/N ratio of DAPK DNA methylation status and its mRNA expression (Fig. 2C).

### Lack of correlation between DAPK protein and mRNA expression

**Figure 3:**
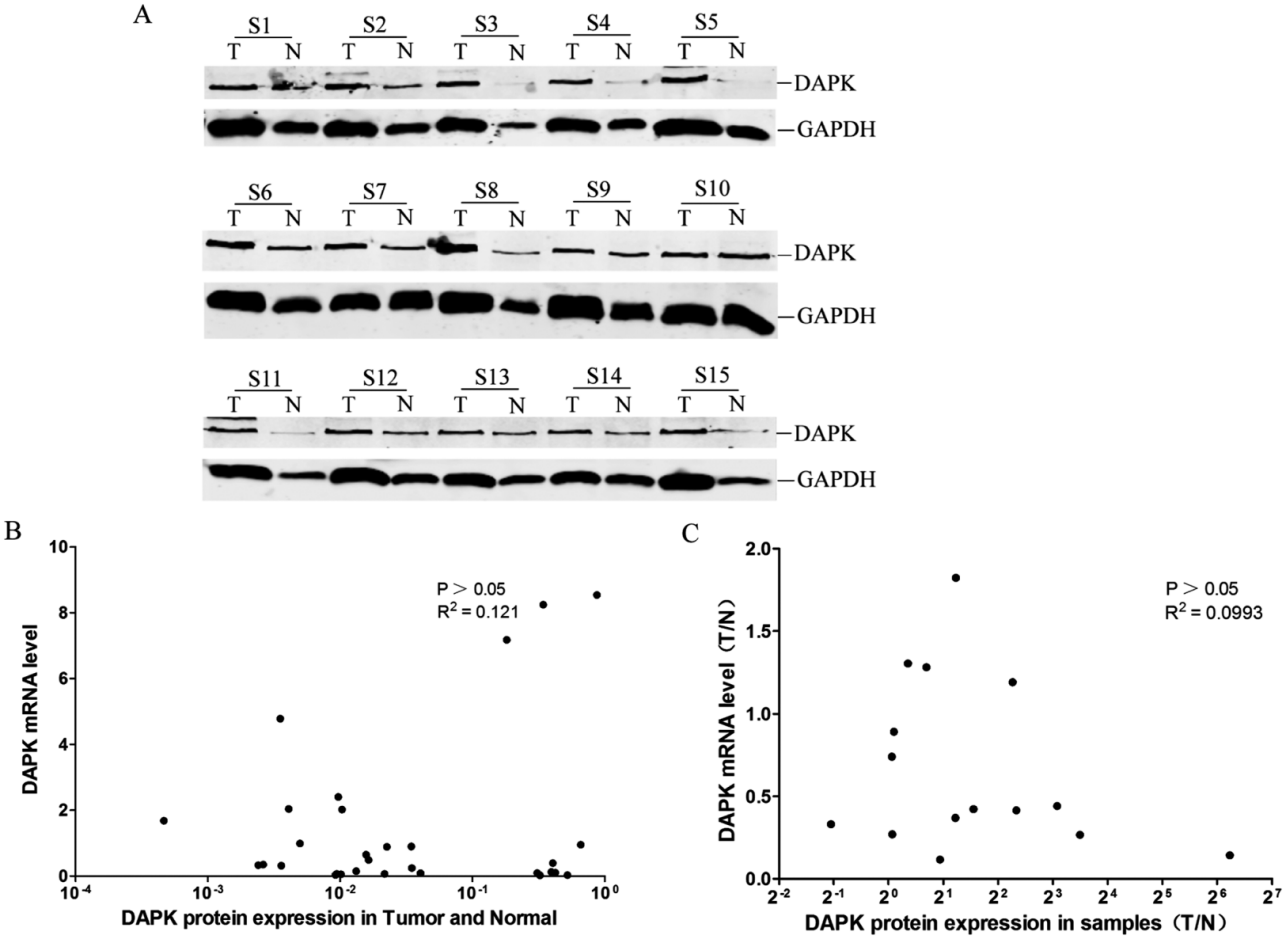
Analysis of the correlation between DAPK mRNA and protein expression in breast cancer samples. (A) The protein expression of DAPK was detected using western blot. T, the breast cancer tissue; N, the corresponding adjacent normal tissue; S1–S15, patient codes. (B) Correlation analysis of *DAPK* mRNA expression and DAPK protein expression in breast cancer samples. The cDNA from HEK293T cells were used as a positive control and formation of the standard curve. (C) Correlation analysis of the T/N ratio of *DAPK* mRNA expression and DAPK protein expression in breast cancer samples.

The DAPK protein expression of these 15 pairs of breast cancer samples was then analyzed using western blot (Fig. 3A). No correlation between the DAPK protein and mRNA expression in the total of 30 samples was observed (Fig. 3B). Moreover, no correlation between the T/N ratio of DAPK protein and mRNA expression of the 15 patient samples was observed either (Fig. 3C).

## Discussion

In this study, we engineered an artificial construct pUC57-methyl to quantitatively measure the DNA methylation rate of DAPK. Using pUC57-methyl, we can resolve the differential affinity problem between the U and M primers and directly compare the proportion of methylated and unmethylated DAPK gene within the same sample. Thus, we can avoid the problem of differential cell composition across the samples and only investigate the changes of the proportion of methylated DAPK gene. However, it was clear that the methylation rate could vary dramatically from one patient to another even in the non-tumor tissues (Fig. 1D). One assumption we need for this type of study is that the tumor and non-tumor samples from the same patient have about the same cell composition. It will be ideal if this study can be performed only in the cancer cell subgroup. Moreover, there were only 15 pairs of breast cancer samples in this study. Although the correlation was very poor among these samples, it cannot be ruled out that a better correlation may be observed in a bigger patient cohort. Future studies using more patient samples will be needed to further confirm the discovery of this study.

As mentioned above, most studies on DAPK DNA methylation used the same set of primers that targets a specific site on DAPK promoter (Katzenellenbogen, Baylin & Herman, 1999). There is no doubt that this site is critical for regulating the transcription of DAPK gene. However, there are multiple CpG islands on DAPK promoter (Benderska & Schneider-Stock, 2014). It is possible that some other sites can also participate in the regulation of DAPK mRNA expression. Moreover, there are no reports on the regulatory element such as enhancer for DAPK gene. The individual status of these transcriptional regulators may also influence the expression of DAPK mRNA. Actually, it was reported before that DAPK protein expression could still be detected at the presence of DNA methylation in non-small lung cancer (NSCLC), renal cell carcinoma (RCC) and chronic lymphoid leukemia (CLL) (Huang et al., 2014; Toyooka et al., 2003), supporting that more components need to be taken into account when interpreting DAPK DNA methylation data.

The catalytic activity of DAPK is regulated by Ca/CaM and by autophosphorylation of Ser-308, which resides within the calmodulin-binding domain (Jin, Blue & Gallagher, 2006; Shohat et al., 2002). Autophosphorylation of Ser-308 prevents calmodulin binding, which is necessary for the kinase activity of DAPK (Jin, Blue & Gallagher, 2006). In this study, we observed a second band a little higher than the DAPK band in some samples (Fig. 3A), which may be the auto-phosphorylated DAPK protein. Further study will be needed to validate this hypothesis.

The protein expression of DAPK is controlled by both ubiquitin–proteasome (Jin et al., 2002; Lee et al., 2010; Zhang, Nephew & Gallagher, 2007) and lysosome pathways (Gallagher & Blue, 2014; Lin et al., 2008, 2009, 2011; Stevens et al., 2009). It is not surprising that the mRNA and protein expression of DAPK did not correlate well (Fig. 3). However, a study showed that hypermethylation of DAPK gene correlated well with DAPK mRNA expression in breast cancer patient samples (Lehmann et al., 2002). The opposite results may be due to the different methods used in measuring DAPK DNA methylation. In our study, pUC57-methyl vector was develop to allow quantitative measurement the DNA methylation rate of DAPK.

However, protein expression is the endpoint of gene expression. If the DNA modification or mRNA levels of DAPK cannot correlate with its protein level, extra caution was needed for interpreting the correlation between their correlations with clinical and pathological parameters as they may actually reflect the activity or expression of the regulatory components rather than the actual DAPK protein expression.

## Supplemental Information

10.7717/peerj.3084/supp-1 Supplemental Information 1 Raw data of Fig. 3.

## Additional Information and Declarations

### Data Deposition

The following information was supplied regarding data availability: The raw data has been supplied as Supplemental Dataset Files.
