# Supplementary figures and images for: Quantitative and correlation analysis of the DNA methylation and expression of DAPK in breast cancer
Source: PeerJ. 2017 Mar 14;5:e3084. doi: 10.7717/peerj.3084 (PMC5354070; doi:10.7717/peerj.3084)

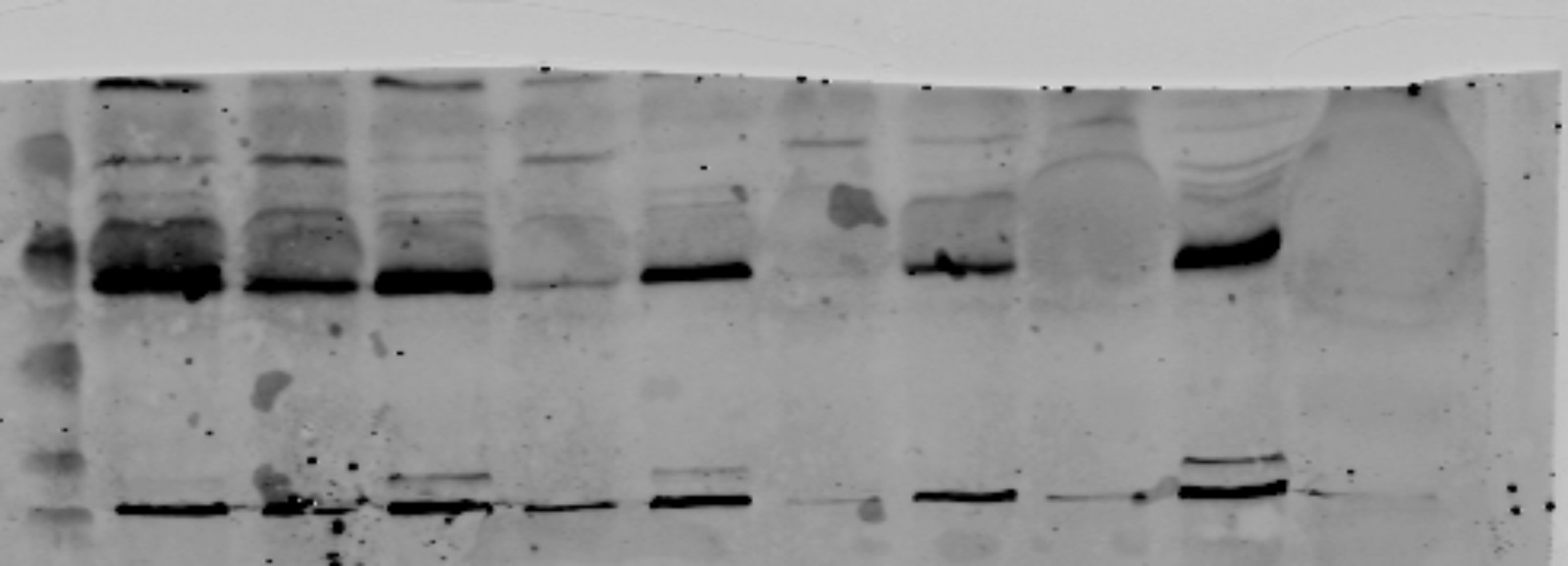

Supplement: Supplemental Information 1 [file peerj-05-3084-s001.zip › Raw dates of fig 3/S1-S5/S1-S5 DAPK.jpg]

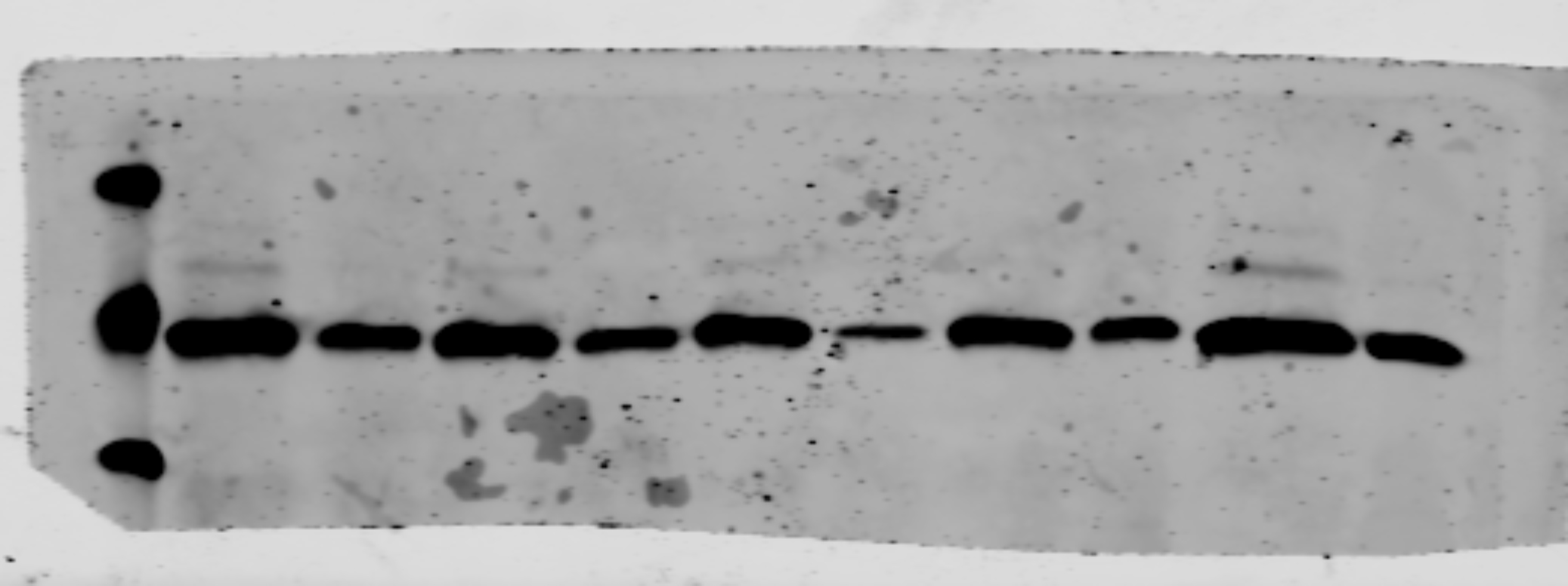

Supplement: Supplemental Information 1 [file peerj-05-3084-s001.zip › Raw dates of fig 3/S1-S5/S1-S5 GAPDH.jpg]

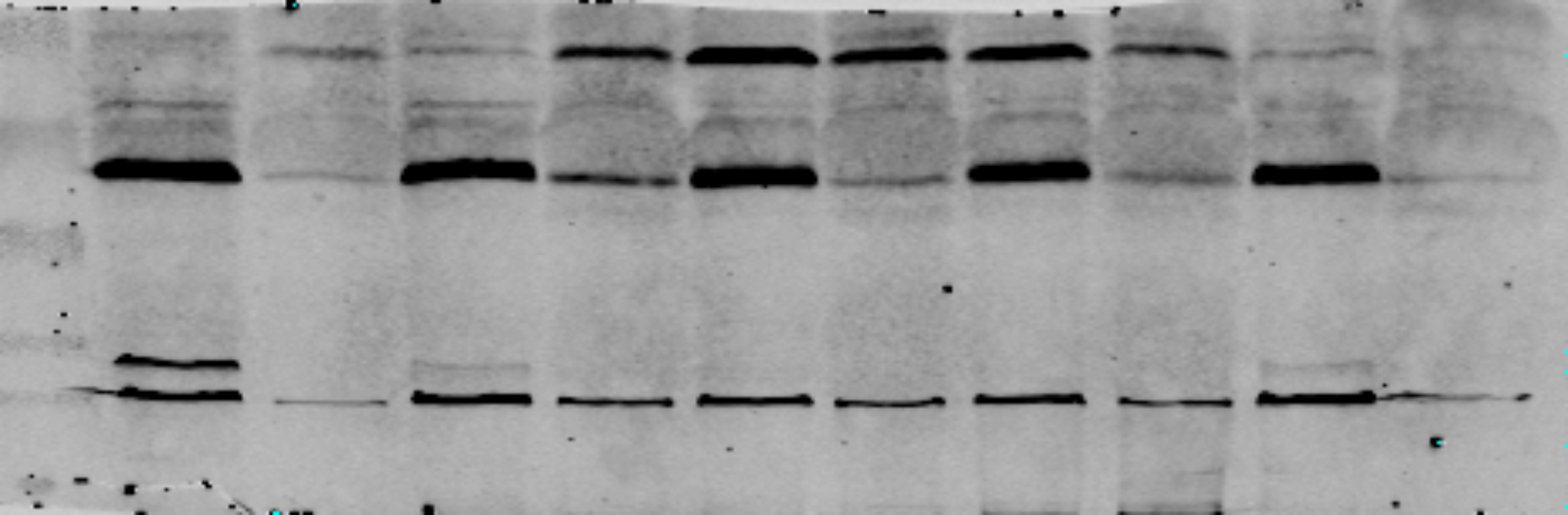

Supplement: Supplemental Information 1 [file peerj-05-3084-s001.zip › Raw dates of fig 3/S11-S16/S11-S16 DAPK.jpg]

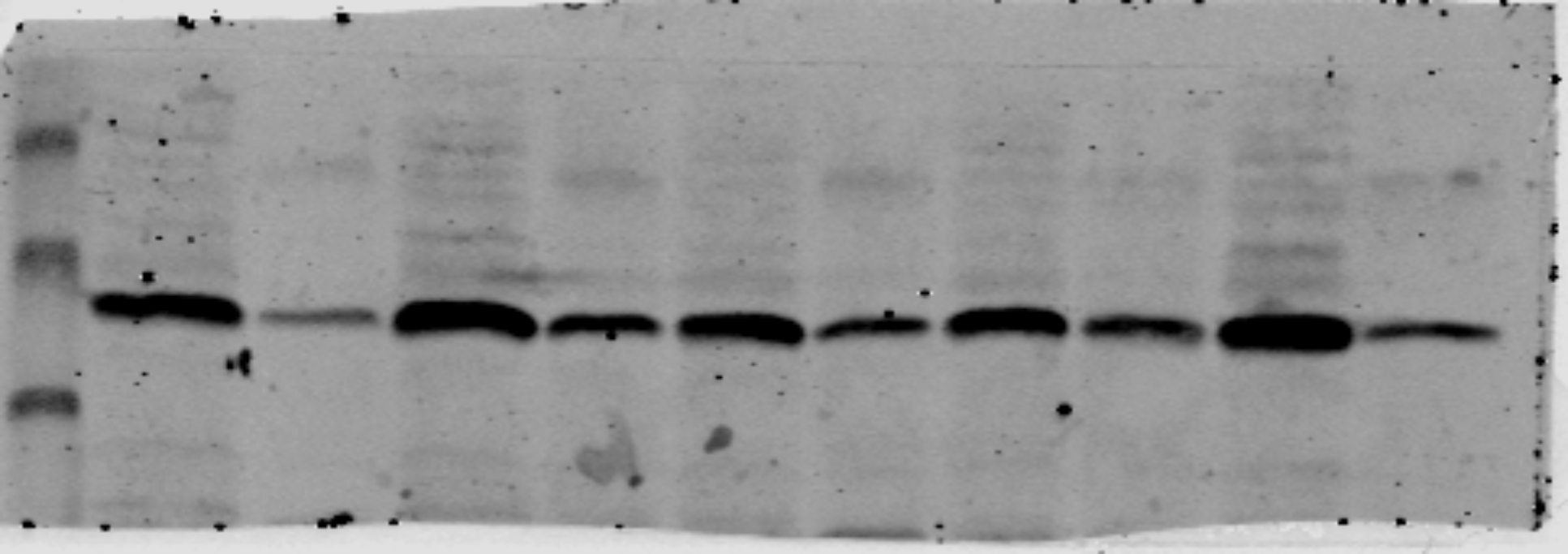

Supplement: Supplemental Information 1 [file peerj-05-3084-s001.zip › Raw dates of fig 3/S11-S16/S11-S16 GAPDH.jpg]

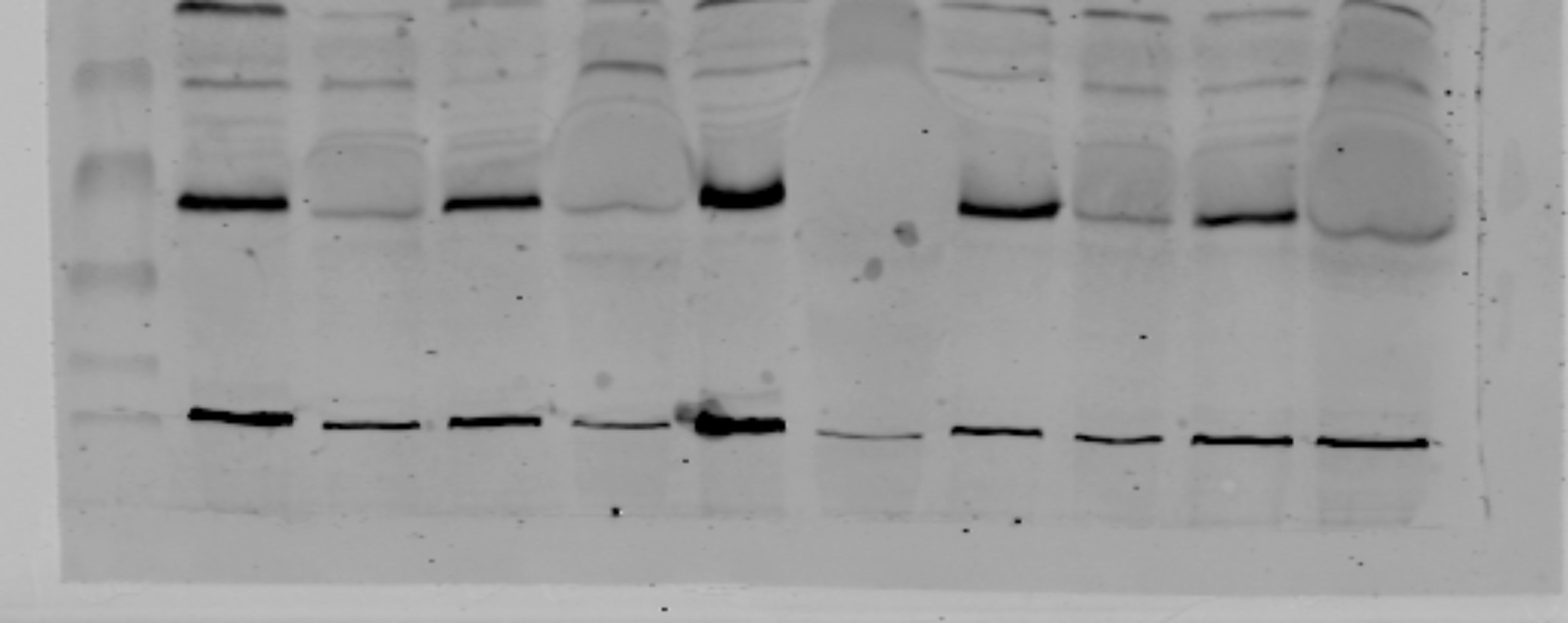

Supplement: Supplemental Information 1 [file peerj-05-3084-s001.zip › Raw dates of fig 3/S6-S10/S6-S10 DAPK.jpg]

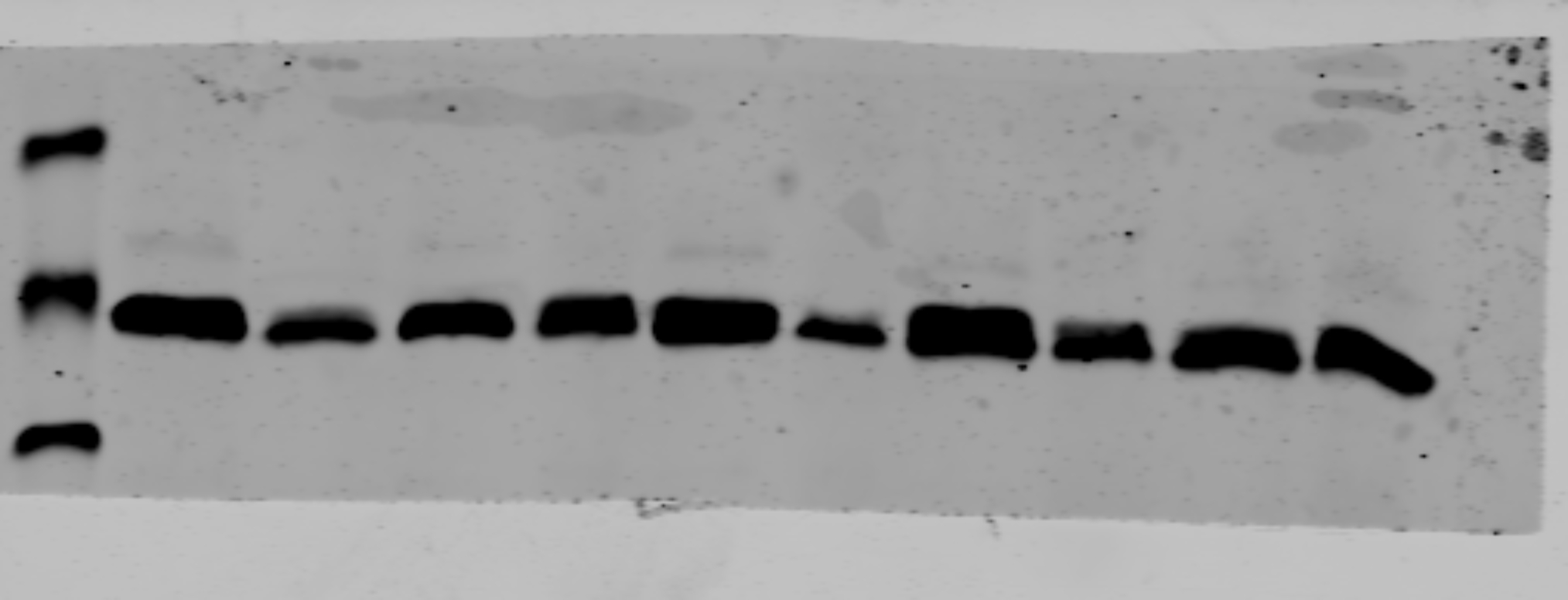

Supplement: Supplemental Information 1 [file peerj-05-3084-s001.zip › Raw dates of fig 3/S6-S10/S6-S10 GAPDH.jpg]
